# Effectiveness of Resistance Exercise on Cognitive Function in Animal Models of Alzheimer Disease: A Systematic Review and Meta-Analysis

**DOI:** 10.14283/jpad.2024.75

**Published:** 2024-04-10

**Authors:** F. O. de Andrade Santos, A. A. Passos, Ricardo Mario Arida, L. Teixeira-Machado

**Affiliations:** 1 Graduate Program in Applied Health Sciences, Federal University of Sergipe, Lagarto, Brazil; 2 Graduate Program in Psychology, Federal University of Sergipe, São Cristóvão, Brazil; 3 Department of Physiology, Federal University of São Paulo, São Paulo, SP Brazil; 4 Department of Education in Health, Federal University of Sergipe, Lagarto, Brazil

**Keywords:** Alzheimer disease, resistance exercise, memory, and anxiety

## Abstract

**Aim:**

Alzheimer’s disease (AD) is among common cause of dementia. Complementary therapies, such as resistance exercise (RE), have been proposed as an alternative for the treatment of AD. We performed a systematic review and meta-analysis to investigate the effects of RE on the cognitive function of AD animal models and their physiological mechanisms.

**Methods:**

This review was submitted to PROSPERO (CRD42019131266) and was done according to PRISMA checklist. Four databases were used in the search: MEDLINE/PUBMED, SCOPUS, Web of Science and Google Scholar. We used SYRCLE and CAMAREDES to assess the risk of bias and methodological quality. We calculated the standardized mean difference using 95% confidence intervals and considered the random effects model and p < 0.05 to determine significance.

**Key Findings:**

A total of 1,807 studies were founded, and after the selection process, only 11 studies were included in this review and 8 studies were included for meta-analysis. Four studies applied RE before AD induction, 7 studies applied RE after AD induction or in the AD condition. All studies included 550 adult and older animals weighing 25–280g. Our analysis revealed that RE had a positive effect on memory in AD animal models but did not show a significant impact on anxiety.

**Conclusion:**

RE performed four or six weeks, more than three days a week, had a significant protective effect on memory. The included studies had a high risk of bias and moderate methodological quality. Therefore, RE can be a potential strategy for preventing cognitive decline in animal models.

**Electronic Supplementary Material:**

Supplementary material is available for this article at 10.14283/jpad.2024.75 and is accessible for authorized users.

## Introduction

**D**ementia affects approximately 55 million people worldwide, and by 2050, these numbers may triple, especially in underdeveloped and middle-income countries (1). One of the main causes of dementia is Alzheimer’s disease (AD), a progressive neurodegenerative disease that leads to selective neuronal cell death and tissue loss throughout the brain (2). The presence of amyloid beta (Aβ) protein deposits, hyperphosphorylation of tau protein with the formation of tangles, neuronal injury, and neurodegeneration leading to memory deficits characterizes the AD (3–5). Furthermore, neurogenesis is also affected by this pathological condition (6). These changes and deficits impair cognition and synaptic plasticity in the central nervous system (CNS) (6, 7).

Although pharmacological intervention is the treatment of choice, non-pharmacological therapies, such as physical exercise, have been proposed as coadjutant therapy for AD treatment (2). The latter has received attention as a form of prevention for cognitive decline because it appears to reduce cognitive and psychological symptoms, in addition to improving the quality of life (QoL) and daily activities (2, 8). For instance, studies have demonstrated that aerobic exercise exerts a positive effect in reducing the rate of progression of cognitive decline in humans with dementia or mild cognitive impairment (9, 10). Furthermore, exercise can induce neurogenesis, improve cognition and memory, and increase the levels of brain-derived neurotrophic factor (BDNF) and interleukin-6 (IL-6) in AD mouse model (6). Among several physical exercise interventions, resistance exercise (RE) has constantly been recommended for older people (11). This approach has been extensively studied in humans, as it is associated with better global cognitive function, memory, and executive function, as well as gain in muscle strength, decreased risk of falls, and changed psychological symptoms such as anxiety and depression (12, 13, 14, 15). Positive effects of RE on cognitive function have also been observed in older people and AD patients (14, 15, 16).

Animal models of AD have been used to study the pathomechanisms and to propose therapeutic strategies for this disease (17). Most investigations conducted in AD animal models have applied aerobic exercise to understand the beneficial effects of exercise in this condition (18, 19). Insights into the effects of RE will be of great value to understand how it could interfere with cognitive functions in the affected brain. Although a recent review highlighted the impact of RE on cognitive dysfunction and AD in both human and animal models (20), to better understand the effectiveness of RE on cognitive function, a comprehensive analysis is needed to cover the cognitive and behavioral aspects of RE in AD animal models. Therefore, we conducted a systematic review and meta-analysis to investigate the effects of RE on the cognitive function of AD animal models and their physiological mechanisms.

## Material and methods

### Registration

This review protocol was registered with the International Prospective Register of Systematic Reviews (PROSPERO) on 31 July 2019 (CRD42019131266). This review was based on the Collaborative Approach to Meta-Analysis and Review of Animal Data from Experimental Studies (CAMARADES) guidelines and compliant with the Preferred Reporting Items for Systematic Reviews and Meta-analyses (PRISMA) (21).

### Eligibility Criteria and search strategy

We define as a guiding question «What is the influence of RE on cognition in experimental animal models?».

The eligibility criteria followed PICOS strategy: P –animal model (rat and mice) with AD; I – only RE before or after brain induction of AD animal models using inclined vertical ladder apparatus and weights; C – sedentary or SHAM; O – cognitive function (primary outcome), anxiety (secondary outcome); and S – preclinical studies.

We considered as inclusion criteria for cognitive and behavioral analysis induced by RE for synthesis analysis the studies that addressed RE before or after brain induction of AD animal models (rat and mice) using inclined vertical ladder apparatus and weights, compared with other interventions (SHAM, control or sedentary), and studies that evaluated cognitive performance. The exclusion criteria were studies that investigated animal models without cognitive impairment, RE associated with nutritional supplements or medications, or that did not address cognitive function. We excluded in vitro studies, studies with humans, and other study types.

We used a specific search strategy in the following databases until June 2023 MEDLINE/PUBMED (we used Syrcle animal filter), SCOPUS, Web of Science (WOS), and Google Scholar, using the keywords «exercise program», «strength training», «resistance training», resistance exercise, “strength exercise”; «cognition», «memory», «cognitive function», “animal”, “rat”, “rodents”, “mice”, “mouse”, and “murine”. The search strategy did not have restrictions on the year or language publication, and we adapted the search strategies according to each database (see details of the search strategy in supplementary content). We manually collected relevant studies from the reference lists of systematic reviews with similar themes found during the search in the databases and from the included studies. Other resources, such as conference proceedings, journals, and other non-bibliographic database sources (conference panel), unpublished and ongoing studies (registered with experimental studies), and free web searches were used in our systematic review.

### Selection and data collection process

Two researchers (FOAS and AAP) independently evaluated the titles and abstracts found through the Rayyan manager (22). We included studies that predefined the eligibility criteria and the PICOS strategy. To facilitate the reading and exclusion of studies, we defined some exclusion criteria (1) non-animal model; (2) studies that investigated animal models without AD; (3) no RE protocol; (4) exercise associated with another intervention (supplementation or medication); (5) not be experimental studies; and (6) measures that did not address cognitive function, memory, or anxiety. We excluded studies that did not meet these criteria and were not related to the research problem. After the evaluation of titles and abstracts, the same two reviewers accessed and read the other full studies and selected them based on the eligibility criteria. After discussion in each phase, the other two researchers (LTM and RMA) resolved the disagreements and discrepancies. The Kappa test was used to verify the inter-rater reliability where values ≤0 indicated no agreement and values 0.81–1.00 indicated perfect agreement (23).

We used Windows Excel® spreadsheet and Review Manager (version 5.4) to perform data extraction (24). The performed tables contained information of the first author’s name, year of publication, country of orign, sample size (control group and intervention group), comparison conditions, number of experimental groups, animal (sex, models, weight, age, drug), protocols and interventions (dose, frequency, duration, equipment), time point measured, outcome measurements, and results. If the data were not available in the table or results section or did not report relevant numerical outcome data in the text, we contacted the authors of these studies by e-mail, or we extracted the data from figures and graphs, when possible. Two researchers (FOAS and AAP) performed this phase, and in the case of discrepancies, the other two researchers (LTM and RMA) resolved them. We contacted eligible authors by e-mail to provide missing or additional data to extract and the information studies.

### Study risk of bias assessment

Two investigators (FOAS and AAP) used SYRCLE and CAMAREDES to assess the risk of bias (RoB) and methodological quality, and two other investigators (LTM and RMA) resolved disagreements (25, 26). SYRCLE consists of six biases analysis (selection, performance, detection, attrition, reporting bias, and other bias) that are identified based on empirical evidence and theoretical considerations. Domains are classified as low (green), uncertain (yellow), and high (red) RoB. The CAMARADES checklist was answered with yes or no, for the criteria of [1] publication in a peer-reviewed journal; [2] statement of control of temperature; [3] randomization to treatment or control; [4] allocation concealment-blinded induction of AD (i.e., concealment of treatment group allocation at the time of induction of AD); [5] blinded assessment of outcome; [6] appropriate animal species and AD models; [7] adaptation/familiarization to exercise apparatus; [8] sample size calculation; [9] statement of compliance with ethical regulations; and [10] statement regarding possible conflicts of interest.

### Synthesis Methods and Meta-analysis

For the meta-analysis, we used the free and open statistical software Review Manager (Computer program) (24). We calculated the treatment effects of the experimental group (RE) versus the control group (sedentary, control, or SHAM) on cognitive function and anxiety through the standardized mean difference (SMD) using 95% confidence intervals (CI) using measures such as mean, standard deviation (SD) and number of participants in each clinical trial group. If the means and SD were not directly available, we created a transformation formula for the data expressed in standard error (SEx√N) and for studies that used graphs to visualize the data, we used the Web Plot Digitizer 4.6 (27).

The criterion adopted for calculating the weights of each study in the meta-analysis is the inverse variance, which for the random effects model considers both the individual standard deviations and the variance between studies, generally calculated using the Der Simonian-Laird method (28). After analysis, data such as effect sizes (ES), 95% CI and p-values were grouped in a table and the results were represented in the Forest Plot, using p < 0.05 to determine significance. We considered the effect size as small (0.2), moderate (0.5), and large (0.8) classified by Cohen et al. (29).

We performed heterogeneity analysis using the chi-square test and the Higgins index (I2), and results with I2<40% indicate non-significant heterogeneity; I2 between 30 and 70% may represent moderate heterogeneity; I2 between 75 and 100% is a significant heterogeneity (28). We performed subgroup analysis to explore heterogeneity.

For sensitivity analysis, we systematically removed one study from analysis to assess the influence and effect of each study on the results. If a study affected or influenced the effect, we excluded it from the meta-analysis. If more than 10 studies were included in the analysis, reporting bias would be evaluated by the asymmetry of the funnel plot.

## Results

### Study selection

**Figure 1:**
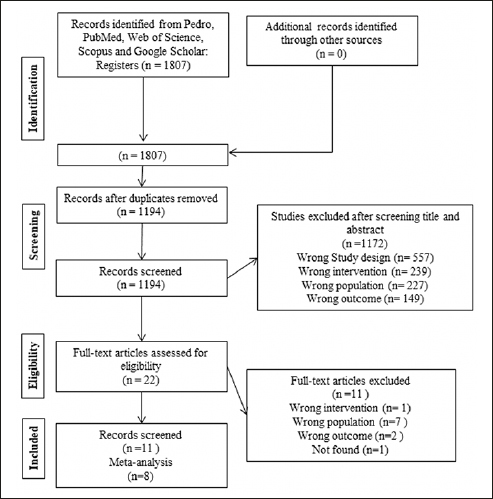
A flow diagram of the systematic review literature search

We identified a total of 1,807 potentially relevant studies in four databases. We removed duplicates (n=613) remaining 1,194 studies. After reading the titles and abstracts, we excluded 1,172 studies and selected 22 for full reading. We excluded 11 studies because they did not meet the eligibility criteria, eight of which were of the wrong population (30–37), one presented wrong outcomes (38), one performed wrong intervention (39), and one study was not found (40). We included in this review the remaining 11 studies that met the inclusion criteria and used different instruments and ways of presenting the same outcome (7, 19, 41–49). For the meta-analysis, we excluded 2 studies (44, 47), one (44) after sensitivity analysis, and another (47) because overlapping population with study in 2019 (46). The Kappa test (1.0 in the first step and 1.0 in the second step) indicates an almost perfect agreement between the researchers. Details of the study selection process are shown in Figure 1.

### Study characteristics

This review included 11 studies published in journals from 2017 to 2023. Of these, four studies applied RE before AD induction (41, 45–47), whereas 7 studies applied RE after AD induction or in the AD condition (7, 19, 42–44, 48, 49). Studies that met the eligibility criteria included 550 adult and older animals weighing 25–280g. Eight studies did not report the weight (7, 41, 42, 44,46–49) and three studies did not report the number of animals (44, 47, 48). All details are shown in Table 1.

**Table 1:** Characteristics and outcomes of included studies

	**Animal**											**Outcomes**		
**1st Author (year)**	**N**	**N by group**	**Model (mice)**	**Sex**	**Age/weight (month/g)**	**Exercise regime**	**Total duration**	**Frequency-Day/week**	**Dose (sets/repetitions)**	**Drug**	**Assessment**	**Memory**	**Anxiety**	**Conclusion**
Resistance exercise after induction AD condition														
Campos (2023) Brazil	-	12–15/group	Transgenic AD	M	6–7 month/not informed	Climbing apparatus is a 110 cm high and 18cm wide ladder, with 2 cm between each step, with an incline of 80o and a shelter at the top	4 weeks	3 days/week	1 set with 11 repetitions in the first two sessions 75% body weight, third and fourth trials was 90%, fifth and sixth trials the overload was 100% body weight, and from the seventh trial on, the increase in load was 3g	Transgenic AD APPswe/PS1dE9	NOR and open field test	No difference between groups in the discrimination index for the novel object recognition test, but improved anxiety symptoms	RE prevented the occurrence of behaviors such as increased locomotion and decreased the percentage of central crossings	Resistance exercise may contribute to the decrease in hippocampal Aβ plaques, and this may have contributed and normalized to memory and behavior on alternate days for 4 weeks in the transgenic AD model
Farzi (2018) Iran	50	Control (10); Sham surgery (10); Aβ+sedentary (10); Aβ+AE (10); Aβ+RE (10)	Wistar	M	2 month/250–280 g	Resistance training (climbing a ladder - 100 cm length, 2 cm grid, 85° incline with weights attached to their tails)	8 weeks	2 days/week	1 set of 10 repetitions	Aβ	NOR	RE significantly increased the novel object exploring time in the Aβ-injected rats	-	Exercise improves AD-induced memory impairment.
Kelty (2019) USA	-	10–14 rats/group (VEH; LPS; LPS-RE)	Wistar	F	1 month / not informed	Progressive resistance training (climbing with weight on vertical ladder)	6 weeks	3 days/week	8 sets	LPS	Barnes Maze Testing	RE was able to reverse spatial learning deficits induced by LPS using the procedure and parameters of the present experiments	-	The improved cognition was associated with increased synaptic plasticity, as well as increased IGF-1/AKT signaling
Liu (2020) China	31	Sedentary AD (7); RE AD (8); Sedentary control (8); RE control (8)	Transgenic AD	M	9 months/not informed	RE climbing up a 1-m ladder 15 times, with progressively heavier weights attached to their tails and a 2-min rest between each climb	4 weeks	Alternate days	1 set of 15 repetitions. The weights that were loaded were equivalent to 15%, 30%, 50%, and 75% of their body weight in weeks 1, 2, 3, and 4, respectively	Transgenic AD (B6; 129-Psenltm-1Mpm Tg (APPSwe, tauP301L)	NOR and open field test	RE increased the recognition index in the NOR test, and reduced the escape latency and the number of errors in the Y-maze test, but did not improve anxiety	RE did not reduce anxiety symptoms induced by Aβ	Resistance training may represent an alternative exercise strategy for delaying disease progression in Alzheimer’s disease
Martini (2020) Brazil	26	Sham surgery (9); STZ (8); STZ + RE (9)	Swizz	M	Adult/25–35g	1-m ladder, with 1.5 cm grids	4 weeks	5 days/week	1 set of 10 repetitions, week 1: adaptation period; week 2:10% body weight; week 3:20% body weight; week 4: 40% body weight; week 5: 60% body weight	STZ	MWM	RE increase in the time spent and the distance travelled in the target quadrant in the MWM test	-	Strength exercise suppressed memory impairment and modulated the hippocampal BDNF/ERK-CAMKII/CREB signalling pathway
Özbeyli (2017) Turkey	64	8 rats/subgroups (Sham: Sedentary; AE; RE; Combined. OVX+D-GAL: Sedentary; AE; RE; Combined)	Wistar	F	6–7 months/260–280 g	Progressive resistance training (climbing with weight on vertical ladder-1.1 m × 0.18 m, 2-cm grid, 80° incline)	6 weeks	3 days/week	8 sets	D-Gal	OR and hole plate test	RE improved working memory in AD-like model	RE did not reduce anxiety symptoms induced by Aβ	The decreased cognitive functions in AD-like model were improved via all exercise type, additionally the increased anxiety-like behaviour in AD-like conditions was not inhibited with RE
Rahmati (2023) Iran	48	Control (12); control-RE (12); AD-Control (12); AD-RE (12)	Wistar	M	2 months / not informed	1-m ladder with 26 rungs and inclined at 85° with a house chamber (20×20×20 cm) placed at the top	4 weeks	3 days/week	5 sets of 4 repetitions. 65, 75, 85, 95, and 100% of the rat’s previous MCL (75% of their body mass, and 30 g were added for each climb repetition)	Aβ	MWM	The RE group showed improvement in learning between trials from days 1 to 4, decreased escape latency, improved the time spent in the target quadrant, and increased the number of crossings across the platform area compared with the control group	–	RE might serve as an adjunct therapy in cognitive function in the AD model
Resistance exercise before induction AD condition														
Lima (2022) Brazil	62	Control (8); Aβ (9); AE (7); AE+Aβ (8); RE (7); RE+Aβ (8); Concurrent (7); Concurrent+Aβ (8)	Wistar	M	2 months / not informed	A vertical ladder custom-made in wood and iron (1.1 × 0.18 m, 2 cm grid, 80° inclination) with a housing chamber (20 × 20 × 20 cm) placed at the top	8 weeks	3 days/week	8 ladder climbs being 2 repetitions for each load, resulting in 8 sets of 8–12 repetitions with a 1-min rest interval between the repetitions	Aβ	OR; SRT; EPM	RE did not prevent the recognition memory deficit induced by Aβ	RE did not reduce anxiety symptoms induced by Aβ	Concurrent training, including running and RE with in the same session, is ineffective in preventing recognition memory deficits in the Aβ rats
Prado Lima (2018) Brazil	80	Control (20); EE (20); RE (20); SE (20)	Wistar	M	3 months/350–380 g	1-m ladder with steps separated by 2 cm from one another	8 weeks	3 days/week	1 set of 8 repetitions	Aβ	OR; SRT; EPM	RE prevents impairments in object recognition memory and social recognition memory deficits and is more effective in memory protection	RE did not reduce anxiety symptoms induced by Aβ	EE and RE cause a reversion of memory deficits and the effect of Aβ on lipid peroxidation induced by Aβ
Schimidt (2019) Brazil	64	8–12/group (control; Control+ AE; Control+ RE; Aβ; Aβ+ AE; Aβ+ RE)	Wistar	M	2 months / not informed	Progressive resistance training (climbing with weight on vertical ladder-1.1 m × 0.18 m, 2-cm grid, 80° incline)	8 weeks	3 days/week	8 ladder climbs with 2 repetitions for each load of 50%, 75%, 90%, and 100% of MCL, 8 sets of 8–12 repetitions with a 1-min rest interval between the repetitions	Aβ	OR; SRT; EPM	RE prevented LTM and STM deficits in OR and SR memory	RE did not reduce anxiety symptoms induced by Aβ	RE and running training have different effects on memory in an Aβ toxicity-induced AD-like model. While running improves social recognition memory, strength training enhances both social recognition and short- and long-term object recognition memory
Schimidt (2021) Brazil	-	10-12/group (Control; Control+GT; Control+ RE; Control+GT+ RE; AD; AD+ +GT; AD + RE; AD+GT+ RE)	Wistar	M	2 weeks/ not informed	Vertical ladder of 110 cm height, 18 cm width, 2 cm between the steps, and 80° inclination with housing chamber (20 × 20 × 20 cm) at the top	8 weeeks	3 days/week	8 ladder climbs with 2 repetitions for each load of 50%, 75%, 90%, and 100% of MCL, 8 sets of 8–12 repetitions with a 1-min rest interval between the repetitions	Aβ	OR; EPM	RE prevented memory and preserved social recognition memory	RE did not prevent anxiety symptoms induced by Aβ	RE or green tea supplementation can be effective against cognitive impairments resultant of an AD-like model, but benefits do not add up when the two interventions are associated

Abbreviation: VEH= Vehicle injected; LPS= lipopolysaccharide; RE= resistance exercise; AE= Aerobic exercise; Aβ= beta-amyloid; D-Gal= D-galactose; OVX= ovariectomized; EE= environmental enrichment; SE= social enrichment; GT= green tea; F= female; M= male; NOR= Novel object recognition; MWM= Morris Water Maze; OR= Object recognition memory task; EPM= Elevated plus maze; SRT= Social recognition task; MCL= maximal carrying load; IGF-I= insulin like growth factor-I; BDNF= brain derived neurotrophic factor

#### Age, sex, and animal model

The studies used adult animals with ages ranging from 4–36 weeks and only two of them used female animals (19, 44). Wistar rats were the most common species (19, 41, 44–47) followed by transgenic AD mice (7, 48) and swizz mice (42). The studies used five different ways to generate neurodegeneration and develop AD models in rodents: beta-amyloid (Aβ) (41, 43, 45–47, 49), lipopolysaccharide (LPS) (44), D-galactose (19), transgenic AD (7, 48), and streptozotocin (STZ) (42). See details in Table 1.

#### RE protocol

All studies used progressive resistance training as RE with climbing with weight on a vertical ladder. The period of training ranged from 6 to 8 weeks, 2 to 5 days per week. The exercise dose ranged from 1 to 8 series with 4 to 12 repetitions. The comparison conditions were aerobic exercise, SHAM, control, sedentary, and social enrichment. See details in Table 1.

### Results of individual studies

#### Body mass and body weight

Three studies evaluated muscle mass (19, 44, 49). These studies used RE after AD induction and reported increased lean muscle mass of muscles such as the gastrocnemius (44, 49), tibialis anterior, and extensor digitorum longus (44), in addition to increasing the cross-sectional area of Myosin heavy chain (MyHC) IIb fibers (49).

From four studies that assessed body weight (7, 19, 41, 44), only one performed RE before AD induction (41) with no difference between the groups. Of the investigations that analyzed RE after AD induction, no difference in body weight was observed between groups in one study (44), while in the other two studies (7, 19) they observed body weight increase in the sedentary groups compared with the exercise groups.

#### Qualitative description of cognitive function (memory)

Most studies in this review showed the positive effects of exercise in reducing and preventing cognitive impairment in rats with AD (7, 19, 42–49), regardless of the timing of AD induction. Only one study showed no significant effect of RE on cognitive impairment in relation to the others (41); this study performed RE before AD induction. To evaluate cognitive function, the most common instruments used were the novel object recognition task (7, 19, 41, 43, 45–48), Morris Water Maze (42, 49), and Barnes Maze Testing (44).

#### Qualitative description of anxiety/locomotor activity

Seven studies evaluated anxiety (7, 19, 41, 45–48). Four applied RE before AD induction (41, 45–47) while three used RE after AD induction (7, 19, 48). Anxiety was assessed by the elevated plus maze (41, 45–47), the open field test (7, 48), and the hole plate test (19). Another way to assess anxiety was by weight and body mass, and whether the animal ate or drank water (7). Only Campos et al. (48) showed that RE prevented the occurrence of anxious behaviors. They observed an increase in total locomotion and a decrease in the percentage of centralized crossings by the open field test in animals submitted to RE (48). The other studies did not show improvement in anxiety outcome compared with the other groups (7, 19, 45–47).

Six studies evaluated locomotion and exploratory activities (9, 41, 45–48). Four studies used RE before AD induction (41, 45–47), and two studies used RE after AD induction (7, 48). All studies demonstrated no difference between the RE and control groups.

#### Qualitative description of neurobiological components

The four studies that performed RE before AD induction demonstrated a reduction in oxidative stress by decreasing lipid peroxidation and improving antioxidant capacity by stimulating neuroprotective action in the hippocampus region (41, 45–47), decreasing malondialdehyde levels and increasing neural growth factor levels (19). Four studies evaluated acetylcholinesterase (AchE) (43, 45–47). Three studies evaluated RE before AD induction; two of them showed that RE prevented the decrease in AchE activity (46, 47), and one (45) did not find significant differences between groups. One study that performed RE after AD induction showed a positive effect (43).

The included studies that performed RE after AD induction reported other significant results (7, 19, 42, 44, 48, 49). RE reduced corticosterone levels (48) and the number of amyloid plaques in the frontal cortex and hippocampus (42, 48) as well as promoted an anti-inflammatory effect by regulating markers such as Tumor Necrosis Factor alpha (TNF-α) and IL-1 beta and increasing the anti-inflammatory mediator IL-10 in the hippocampus (7). Two studies reported increased levels of Insulin Like Growth Factor (IGF)-1 and increased proliferation of cell nuclear antigen signaling and proliferation in the dentate gyrus (19, 44). Ozbely et al. showed that RE decreased malondialdehyde levels and increased neural growth factor levels (19). RE favored the modulation of BCL-2 associated protein X (Bax) and B-cell lymphoma protein 2 (Bcl2) protein levels and increased levels of presynaptic vesicular proteins (42). Other studies have also shown increased presynaptic vesicular proteins rather than postsynaptic structural proteins (7, 44). Two studies that evaluated BDNF presented controversial results (19, 42). Increased BDNF and tropomyosin kinase B (TrκB) receptor levels were observed in the hippocampus of AD mice after RE (42), while no significant difference was noted in another study (19). The decrease in the number of MyHC IIb fibers and the number of myofibril satellite cells in AD rats was reverted by RE (49).

### Methodological Quality Assessment

We assessed the quality of each study using the 10-item checklist of CAMARADES (26). The criteria comprise (1) publication in a peer-reviewed journal, (2) statement of control of temperature, (3) randomization to treatment or control, (4) Allocation concealment - blinded induction of AD (i.e., concealment of treatment group allocation at the time of induction of AD), (5) blinded assessment of outcome, (6) appropriate animal species and AD models, (7) adaptation/familiarization to exercise apparatus, (8) sample size calculation, (9) statement of compliance with ethical regulations, and (10) statement regarding possible conflicts of interest (26). The average range quality score range for the eleven included studies was 6.9 (range 6–8). All studies were published in peer-reviewed journals. Temperature control and random allocation to groups were described in 10 of the 11 included studies (90.9%), only Farzi et al. (43) did not describe them. All included studies did not report allocation concealment, whereas blinded assessment was documented in four of eleven studies (36.3%) (42, 45–47). Appropriate animal species and the use of adaptation and familiarization with the exercise apparatus were used in all included studies. All included studies did not report how they performed the sample size calculation. A statement of compliance with regulatory requirements was reported in all included studies. A statement of conflicts of interest was reported in seven of the eleven studies (63.6%); only Lima et al. (41) and Schmidt et al. (46) did not report it (Table 2).

**Table 2:** Methodological Quality Assessment using the 10-item checklist of CAMARADES

**1st Author (year)**	**1**	**2**	**3**	**4**	**5**	**6**	**7**	**8**	**9**	**10**	**Quality score**
Farzi (2019)	y	n	y	n	n	y	y	n	y	y	6
Kelty (2019)	y	y	n	n	n	y	y	n	y	y	6
Lima (2022)	y	y	y	n	n	y	y	n	y	n	6
Liu (2020)	y	y	y	n	n	y	y	n	y	y	7
Martini (2020)	y	y	y	n	y	y	y	n	y	y	8
Özbeyli (2017)	y	y	y	n	n	y	y	n	y	y	7
Prado Lima (2018)	y	y	y	n	y	y	y	n	y	y	8
Schimidt (2019)	y	y	y	n	y	y	y	n	y	n	7
Schimidt (2021)	y	y	y	n	y	y	y	n	y	y	8
Campos (2023)	y	y	n	n	n	y	y	n	y	y	6,0
Rahmati (2023)	y	y	y	n	n	y	y	n	y	y	7,0

Legends: (1) publication in a peer-reviewed journal; (2) statement of control of temperature; (3) randomization to treatment or control; (4) allocation concealment- blinded induction of Alzheimer’s disease (i.e., concealment of treatment group allocation at the time of induction of ad); (5) blinded assessment of outcome; (6) appropriate animal species and ad models; (7) adaptation/familiarization to exercise apparatus; (8) sample size calculation; (9) statement of compliance with ethical regulations; and (10) statement regarding possible conflicts of interest.

### Risk of Bias (RoB) of Included Studies

**Figure 2:**
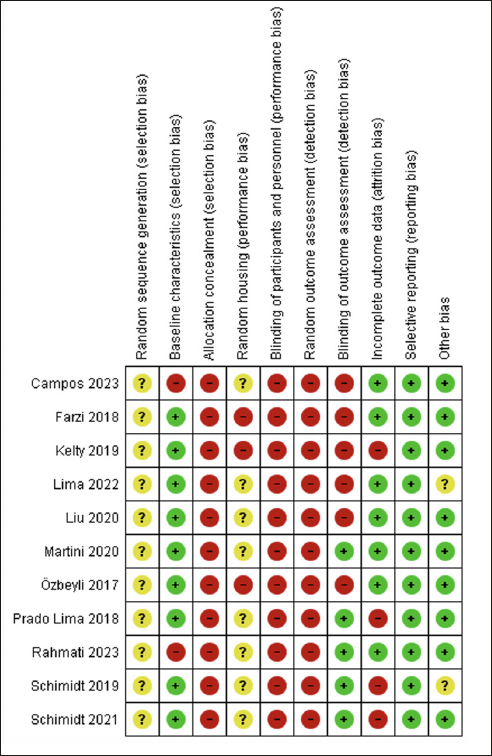
Risk of bias assessment of the included studies by SYRCLE Green = low risk of bias; yellow = unclear risk of bias; red = high risk of bias

The RoB assessment for all included studies is displayed in Figure 2. We considered a high risk of selection bias because no study concealed the allocation, and 7 studies had a high risk of bias considering baseline characteristics (7, 41, 44, 46–49), as they did not report the animal’s weight. Additionally, the studies did not clarify how the random sequence of the groups was conducted; they performed randomization but did not explain the procedure. Among these studies with a high risk of this bias, three (41, 46, 47) performed RE before AD induction.

Concerning performance bias, all studies showed a high risk of bias for blinding caregivers and researchers. Only four studies that performed RE after AD induction showed a high risk for animal housing (19, 43, 44, 48), while the others presented an unclear risk (7, 41, 42, 45–47, 49). We classified the studies with unclear bias, considering that they reported allocating the animals to cages but did not explain the process.

Detection bias was assessed by blinding the outcome assessors and randomizing the outcome assessment. Four studies (42, 45–47) performed blinding, of which only study (42) conducted RE after AD induction. No study reported randomization to assess outcomes.

For attribution bias, we classified four studies (44–47) as having a high risk of bias because they did not specify the number of animals in each group (i.e., 8–10 per group) and did not report on the possible loss of animals and potential deletion of analysis data; of these studies only Kelty et al. performed RE after induction AD (44). We classified the other 7 studies as having a low risk of bias (7, 19, 41–43, 48, 49), and only the study by Lima et al. conducted RE before AD induction (41).

For reporting bias, we classified all studies as having a low risk of bias because they presented all the outcomes that were assessed. Regarding other possible sources of bias, we assessed whether the study was free from inappropriate influence from funders. Two studies (41, 46) that conducted RE before AD induction was classified as having an unclear risk of bias because they did not make it clear, and the other studies (both before and after AD induction) were classified as having a low risk of bias (7, 19, 42–45, 47–49).

### Synthesis of results (meta-analysis)

#### Inclusion criteria in meta-analysis

Of the 11 studies included in our review, we considered 9 studies for the meta-analysis. Among these, we included 7 studies in the effect analysis of RE on recognition memory (19, 41–44, 48, 49), and 5 studies in the analysis of anxiety (7, 19, 41, 45, 46). We excluded two studies from the analysis of recognition memory because they did not present the total result of the test, dividing it into long- and short-term memory (45, 46). Due to sensitivity analysis, Campos et al. (48) was excluded from the anxiety analysis due to its different method of measuring.

There were not enough studies to perform analyzes on social recognition memory, AchE, and RE before induction because two of the studies (45, 46) presented data differently from the others (41). Consequently, we included the study by Lima et al. (41) in the overall RE after induction analysis for memory and anxiety outcomes.

#### Effect of RE on cognitive function (memory) and subgroup analysis

**Figure 3:**
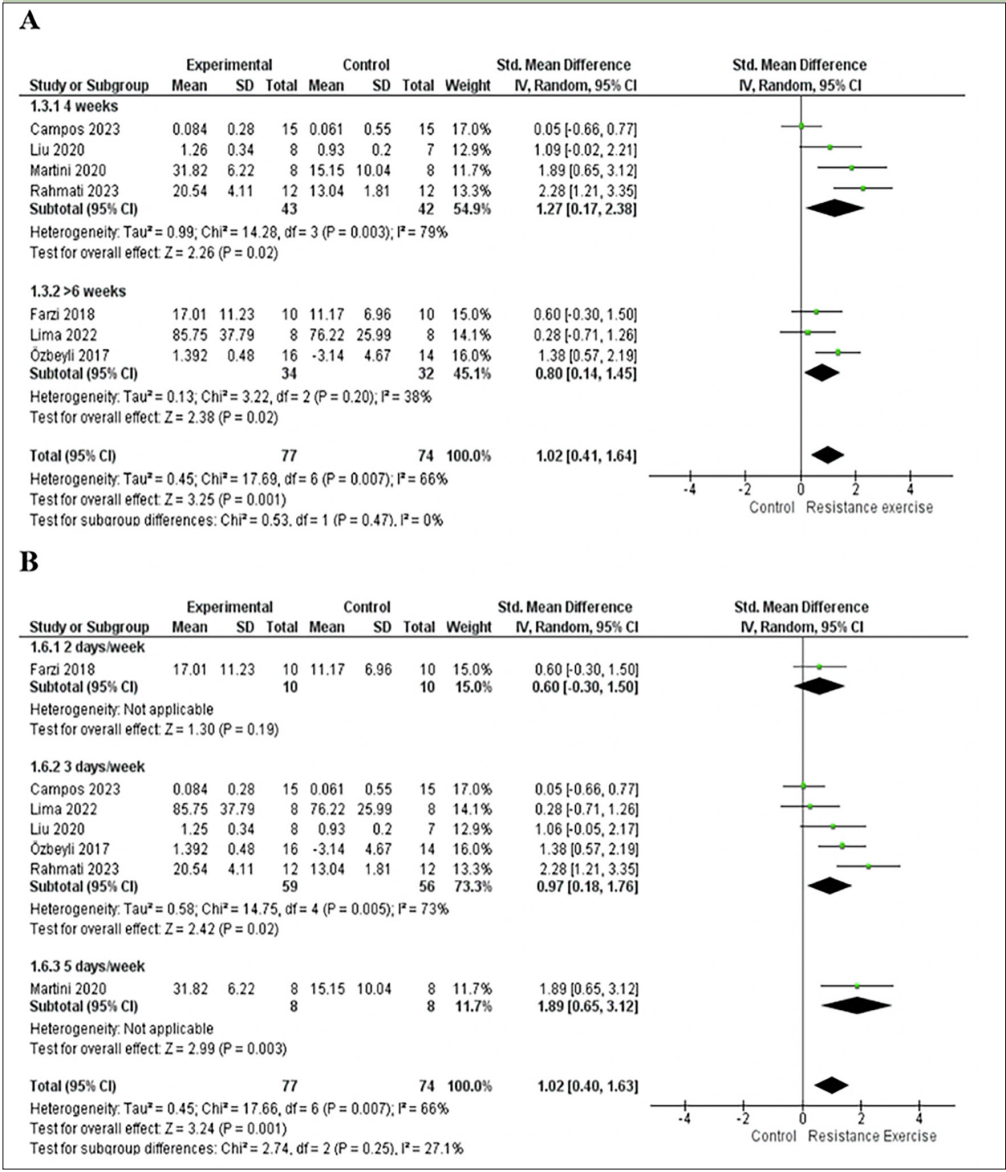
A. Forest plot of the intervention time subgroup analysis of the effect of RE on memory compared with the control group. B. Forest plot of the intervention frequency per week subgroup analysis of the effect of ER on memory compared with the control group Random effect model was applied. Std: standard; CI: confidence interval.

The 7 included studies enrolled 151 animals, with 77 in the RE group and 74 in the control group. In the meta-analysis, we found a significant effect of RE on memory (p=0.001) with a large effect size (ES) (1.02; 95% CI: 0.41, 1.64) and moderate heterogeneity (I2=66%) compared with the control group. Overall, although the standard deviations (SD) of three studies touch the null line (7, 41, 43), they tend to favor the intervention. The study by Campos et al. (48) falls exactly on the null line and has a greater weight (17%) in the analysis, which may partly explain the moderate heterogeneity. To explore heterogeneity, we conducted an intervention time subgroup analysis. Both subgroups, 4-week (p=0.02; I2= 79%; ES= 1.27; 95% CI: 0.17, 2.38) and more than six weeks (p=0.02; I2=38%; ES= 0.80; 95% CI: 0.14, 1.45), exhibited large effect sizes and significant effects. However, the 4-week intervention subgroup displayed high heterogeneity (I2= 79%) and a wide confidence interval (CI). See details in Fig 3A.

For the subgroup analysis based on the frequency of intervention per week, the subgroup with 2 days per week showed no significant effect (p=0.19) and a moderate ES (0.6; 95% CI: −0.30, 1.50), while the subgroup with 5 days per week had a significant effect (p=0.003) and a large effect size (1.89; 95% CI: 0.65, 3.12). It was not possible to analyze heterogeneity, as both subgroups (2 days and 5 days) had only one study. (42, 43). The subgroup with 3 days per week showed a significant effect (p=0.02) and a large effect size (0.97; 95% CI: 0.18, 1.76) but exhibited moderate heterogeneity (I2=73%). See details in Fig 3B.

**Figure 4:**
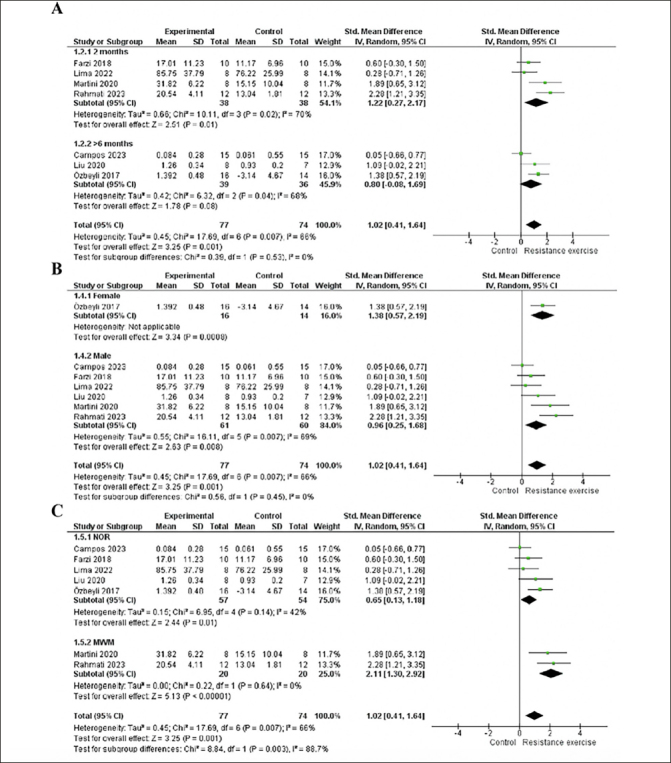
A. Forest plot of age subgroup analysis of the effect of RE on memory compared with the control. B. Forest plot of the sex subgroup analysis of the effect of RE on memory compared to control. C. Forest plot of assessment instruments subgroup analysis of the effect of RE on memory compared with the control Random effect model was applied. Std: standard; CI: confidence interval.

We conducted an age subgroup analysis, including studies with 2-month-older animals, which showed a significant effect (p=0.01), moderate heterogeneity (I2=70%), and large effect size (1.22; 95% CI: 0.27, 2.17). On the other hands, studies involving animals older than six months did not yield a significant effect (p=0.08; I2= 68 %; ES= 0.8; 95% CI: -0.08, 1.69). This result may be attributed to the study by Campos et al. (48), which carries greater weight in the analysis but does not demonstrate a significant effect. See details in Fig 4A.

For the sex subgroup analysis, females exhibited a significant effect (p=0.0008) with a large effect size (1.38; 95% CI: 0.57, 2.19). Heterogeneity assessment was not possible as the group contained only one study (19). In contrast, males showed a significant effect (p=0.008) and a large effect size (0.96; 95% CI: 0.25, 1.68) and moderate heterogeneity (I2=69%). Both subgroups demonstrated a large effect size and a significant effect, but the male subgroup was associated with heterogeneity in the analysis. See details in Fig 4B.

In the subgroup analysis of assessment instruments, the novel object recognition task subgroup demonstrated a significant effect (p=0.01), with a moderate effect size (0.65; 95% CI: 0.13, 1.18) and moderate heterogeneity (I2=42%). Conversely, in the Morris Water Maze subgroup, there was no heterogeneity, accompanied by a large effect size (2.11; 95% CI: 1.30, 2.92) and significant effect (p<0.00001). Both subgroups exhibited a significant effect of the intervention; however, the novel object recognition task subgroup had a moderate effect size and heterogeneity, while the Morris Water Maze subgroup implied a large effect size of the intervention. Please refer to Figure 4C for details.

In the sensitivity analysis, individually withdrawing studies by Martini et al. (42) (p=0.006; I2=67%; ES= 0.91), Ozbeyli et al. (19) (p=0.008; I2= 69%; ES= 0.96), and Rahmati et al. (49) (p=0.004; I2=53%, ES= 0.81) resulted in a reduction in the effect size and the significant effect of the intervention, although they favored the RE. Only the study by Rahmati et al. (49) contributed greater heterogeneity to the analysis. Conversely, removing studies by Lima et al. (41) (p=0.001; I2=69%, ES= 1.15), Farzi et al. (43) (p=0.003; I2=71%, ES= 1.11), and Campos et al. (48) (p<0.0001; I2=52%, ES= 1.21) led to an improvement in ES. The study by Campos et al. (48) contributed greater heterogeneity to the analysis. The study by Liu et al. (7) did not cause any difference in ES (1.02); however, its removal increased heterogeneity (I2=72%).

#### Effect of RE on anxiety and subgroup analysis

**Figure 5:**
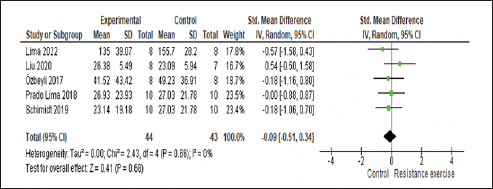
Forest plot of analysis of the effect of RE on anxiety compared with the control group Random effect model was applied. Std: standard; CI: confidence interval.

In the sensitivity analysis, the study by Campos et al. (48) contributed to heterogeneity in the analysis due to its different method of measuring anxiety (number of crossings), and the other studies used time. As a result, it was excluded from the analysis. The five included studies enrolled 114 animals, with 57 in the RE group and 57 in the control group. Our meta-analysis found no significant effect of RE on the anxiety of rats with AD (p=0.22) yielding a small effect size (0.29; 95% CI: −0.76, 0.17) and non-significant heterogeneity (I2=34%) compared with the control group. Please refer Figure 5 for details.

## Discussion

This systematic review and meta-analysis complied evidence investigating the effects of RE on cognitive function and its physiological mechanisms in AD animal models. The 11 included studies explored memory, with four applying RE before AD induction and 7 applying RE after AD induction (7, 19, 41–49). Among these, 7 studies also assessed anxiety, with 4 applying RE before AD induction (41, 45–47) and 3 applying RE after AD induction (7, 19, 48). In general, nearly all studies indicated positive changes in neurobiological components. Our results revealed a positive effect of RE on memory; however, no significant effect on anxiety was observed.

### Effects of RE on memory in AD animal models

Most of the included studies that administered RE both before and after AD induction reported positive effects on memory (7, 19, 42–47, 49). However, the studies conducted by Lima et al. (41) (RE before induction) and Campos et al. (48) (RE after induction) did not demonstrate a positive effect on this outcome. The meta-analysis indicated a significant effect on memory (p=0.001) and exhibited a large effect size (1.02). However, our analysis revealed moderate heterogeneity (66%) compared with the control group. Thus, considering the limited number of such studies and variations in measurement methods, we did not analyze studies that applied RE before AD induction. More precise and standardized studies are required to explore this topic.

Recent reviews have indicated that RE improves cognitive function, specifically memory, attention, and executive function in older people with mild cognitive impairment (20, 50). A randomized clinical trial demonstrated that a RE program in people with mild AD was positively associated with improved cognitive function and better performance in activities of daily living (51). These findings align with the results of our included studies involving AD animal model, suggesting that RE may be a useful tool for the prevention and treatment of cognitive impairment in AD patients.

Sex and age are other important factors to be considered in our analysis. Considering sex differences, this review selected only two studies (19, 44) with female animals. Our analysis showed a higher effect estimate in female animals than in males. This data is relevant because studies of sex and gender differences in AD have reported that women are at greater risk (52, 53), suggesting the need for more studies with female animals to better understand the impact of RE in AD animal models. Another point to be considered is the longer life expectancy for females, and age being the greatest risk factor for AD (53). In our review, only three studies used animals older than 6 months (7, 19, 48), and only one of the included studies used females at this age (19), which limits the number of studies and implies the accuracy of the results. In our meta-analysis, younger animals had a higher effect estimate, despite the heterogeneity between studies, as two studies reached the null line and wide CI. Therefore, starting a regular RE program at a younger age may be considered as a strategy to prevent further cognitive decline.

### Effects of RE on anxiety in AD animal models

Seven out of the 11 included studies in our review did not report a positive effect of RE on anxiety. Existing evidence demonstrates that RE can reduce anxiety in both healthy population and those with chronic clinical conditions (54, 55). Potential mechanisms underlying these benefits include an increase in social participation and the feeling of self-efficacy, along with physiological effects such as an increase in IGF-1 levels, a decrease in inflammation, and modulation of cortisol levels (55, 56).

A preclinical study showed that RE protects against the onset of anxiety/depression by decreasing hippocampal TRκB signaling and neuroinflammation in mice exposed to prolonged stress (56). However, our meta-analysis did not reveal a significant effect on anxiety. Although there was no statistical heterogeneity, the included studies exhibited a high RoB, which may impact reported results due to heterogeneity, such as poorly standardized experimental protocols.

Possible explanations include the use of higher intensities, which can generate greater stress. Therefore, despite anxiety occurring in approximately 39% of people with AD and representing a risk factor for disease development (57), there is a lack of consistent information about the effect of RE on behavioral changes and neuropsychiatric symptoms in the AD population. More standardized clinical and pre-clinical studies, considering different intensities, are needed to investigate the anxiolytic effect of RE in AD and understand its possible mechanisms.

### Physiological mechanisms of the RE in AD animal models

In addition to improving memory, RE has been shown to reduce the number of Aβ plaques (7, 48), tau hypophosphorylation and lipid perioxidation in the hippocampus and cortex (19, 41, 45–47). It also increases the number of microglia (48) and levels of IL-10 (anti-inflammatory mediator) (7). Our review demonstrated that RE decreased proinflammatory factors (TNF-α e IL-1) (7). In the initial stage of the disease, microglia perform an immune response that results in the removal of Aβ through the phagocytosis mechanism, engulfing insoluble deposits of this peptide and activating extracellular proteases, such as neprilysin. However, in the late stages of AD, there is a decrease in the capacity of microglia to phagocytose Aβ, consequently increasing pro-inflammatory cytokines such as TNF-α e IL-1 (58, 59). In this regard, applying RE in the early stages should be a good strategy for reducing neuroinflammation and lipid peroxidation caused by AD.

Our review also demonstrated that RE could reduce malondialdehyde levels, a result of lipid peroxidation (19). Its high level has been considered a risk factor for AD, as it contributes to the pathogenesis of the disease (60). RE can be employed as a modifying factor and a prevention strategy in people with cognitive decline and those with high levels of malondialdehyde. Another mechanism observed in this review was an increase in the levels of presynaptic vesicular proteins, such as synaptotagmin-1, in the frontal cortex and hippocampus (7, 44). These proteins are essential for maintaining the homeostasis of cognitive function, playing fundamental role in synaptic development and serving as a key calcium sensor for exocytosis and endocytosis. Moreover, the reduction of these proteins can induce slow neurodegeneration (61).

Physical exercise has the capacity to increase neurogenesis by regulating neurotrophins such as Brain-Derived Neurotrophic Factor (BDNF), Nerve Growth Factor (NGF) and Insuline-like Growth Factor-1 (IGF-1), primarily in the hippocampus through DNA methylation and gene transcription (62). These neurotrophins play a protective role against oxidative stress caused by neurodegenerative diseases. Our review presented divergent findings regarding BDNF levels among the studies. While the study by Martini et al. (42) observed increased BDNF levels, Ozbeyli et al. (19) found no difference between the RE and control groups. This disparity in results can be attributed to variations in animal models, type of drug used to induce AD, and the timing of sample collection after RE. Physical exercise enhances neuronal plasticity by increasing the signaling of pathways mediated by BDNF through CaMKII, ERK1/2, and CREB proteins, while IGF-1 activates other pathways such as AKT and ERK1/2, leading to increased neuronal proliferation (62, 63). Our review supports these findings and demonstrates that RE can elevate levels of NGF, IGF-1, BDNF and TrκB receptor. This signaling occurs through the mediation of CaMKII, ERK1/2, and CREB proteins, suggesting that RE can activate antiapoptotic mechanisms, promote neurogenesis, and exert a protective effect on neurons, consequently improving cognitive function.

### Impact of intensity and duration of RE

Guidelines for physical exercise suggest that, to enhance muscular fitness in the older people, RE with light to moderate intensity (up to 50% of 1 maximal repetition), and three sets of 8–12 repetitions performed two to three times a week can already elicit muscular and molecular effects/adaptations. Furthermore, it has been suggested that performing more sets (4 sets) per muscle group is more efficient for gaining strength (64, 65).

In our review, studies employed different exercise intensities and regimens depending on the timing of AD induction. Most studies that administered RE before AD induction used exercise with moderate to high intensity, involving 8 sets of 8–12 repetitions (41, 46, 47). Although, studies that implemented RE after AD induction also applied exercise with moderate to high intensity, they did not reach a consensus regarding the number of sets and repetitions. Among the studies that used RE after AD induction, four involved 1 set of 10–15 repetitions (7, 42, 43, 48), two studies used 8 sets (19, 44), and one study employed 5 sets of 4 repetitions (49).

Our meta-analysis revealed that, although both intervention durations (four and six weeks) yielded significant effects on memory, RE lasting more than six weeks provided more reliable results than exercise performed for only four weeks. Additionally, our analysis demonstrated that RE performed more than three days a week had a greater estimated effect, suggesting that a higher frequency of exercise led to a more pronounced protective effect on memory.

### Limitation

The results of this systematic review and meta-analysis must be considered with some limitations. The included studies presented a high risk of bias, mainly in performance and detection; that is, the researchers were not blind, the allocation of animals was not done properly between groups, and they did not describe how the animals were randomized. Another point to be considered is the difference in the moment of AD induction between the included studies, and this also makes the interpretation and implications of the results difficult, as the moment of induction changes the purpose of the exercise, requiring more studies to observe the effects of RE before induction. Our meta-analysis exhibited moderate heterogeneity, attributed to several factors, including sex, age, training frequency, and intervention time.

The included studies differed in terms of animal sex; most studies used male animals, and only two included female animals. Age also varied between studies, with most using adult rather than older animals. The chosen animal model was also limiting, as we did not restrict the type of model. More studies using transgenic models are required. Regarding training, although the intensity was more standardized, the intervention time and the number of sets and repetitions per session were not standardized between included studies. Furthermore, our analysis included only 7 studies for memory outcomes and five studies in anxiety outcomes.

More studies, especially those evaluating anxiety, are needed. All these associated factors, along with possible confounding factors and heterogeneity, prevent a robust quantitative assessment and accurate interpretation of the results. More high-quality pre-clinical studies are needed to confirm the effects of RE in improving cognition/memory and anxiety in AD animal models.

## Conclusion

Our work revealed that RE had a positive effect on memory in AD animal models but did not have a significant impact on anxiety. Therefore, we consider RE a potential strategy for preventing cognitive decline in animal models. However, the included studies exhibited a high risk of bias due to variations in their characteristics. This conclusion underscores the necessity for more standardized studies involving animal models to gain a better understanding of the mechanisms by which RE influences cognitive function.

### Supplementary material

Supplementary material, approximately 332 KB.
